# Fibroblast Growth Factor 1 Reduces Pulmonary Vein and Atrium Arrhythmogenesis *via* Modification of Oxidative Stress and Sodium/Calcium Homeostasis

**DOI:** 10.3389/fcvm.2021.813589

**Published:** 2022-01-18

**Authors:** Yen-Yu Lu, Chen-Chuan Cheng, Shih-Yu Huang, Yao-Chang Chen, Yu-Hsun Kao, Yung-Kuo Lin, Satoshi Higa, Shih-Ann Chen, Yi-Jen Chen

**Affiliations:** ^1^Division of Cardiology, Department of Internal Medicine, Sijhih Cathay General Hospital, New Taipei City, Taiwan; ^2^School of Medicine, College of Medicine, Fu-Jen Catholic University, New Taipei City, Taiwan; ^3^Division of Cardiology, Chi-Mei Medical Center, Tainan City, Taiwan; ^4^Division of Cardiac Electrophysiology, Cardiovascular Center, Cathay General Hospital, Taipei, Taiwan; ^5^Post-Baccalaureate Medicine, College of Life Science, National Tsing Hua University, Hsinchu City, Taiwan; ^6^Department of Biomedical Engineering, National Defense Medical Center, Taipei, Taiwan; ^7^Graduate Institute of Clinical Medicine, College of Medicine, Taipei Medical University, Taipei, Taiwan; ^8^Department of Medical Education and Research, Wan Fang Hospital, Taipei Medical University, Taipei, Taiwan; ^9^Cardiovascular Research Center, Wan Fang Hospital, Taipei Medical University, Taipei, Taiwan; ^10^Division of Cardiology, Department of Internal Medicine, School of Medicine, College of Medicine, Taipei Medical University, Taipei, Taiwan; ^11^Cardiac Electrophysiology and Pacing Laboratory, Division of Cardiovascular Medicine, Makiminato Central Hospital, Okinawa, Japan; ^12^Heart Rhythm Center and Division of Cardiology, Department of Medicine, Taipei Veterans General Hospital, Taipei, Taiwan; ^13^Cardiovascular Center, Taichung Veterans General Hospital, Taichung, Taiwan

**Keywords:** atrial fibrillation, calcium regulation, fibroblast growth factor 1, oxidative stress, pulmonary vein

## Abstract

**Rationale:**

Atrial fibrillation is a critical health burden. Targeting calcium (Ca^2+^) dysregulation and oxidative stress are potential upstream therapeutic strategies. Fibroblast growth factor (FGF) 1 can modulate Ca^2+^ homeostasis and has antioxidant activity. The aim of this study was to investigate whether FGF1 has anti-arrhythmic potential through modulating Ca^2+^ homeostasis and antioxidant activity of pulmonary vein (PV) and left atrium (LA) myocytes.

**Methods:**

Patch clamp, western blotting, confocal microscopy, cellular and mitochondrial oxidative stress studies were performed in isolated rabbit PV and LA myocytes treated with or without FGF1 (1 and 10 ng/mL). Conventional microelectrodes were used to record electrical activity in isolated rabbit PV and LA tissue preparations with and without FGF1 (3 μg/kg, i.v.).

**Results:**

FGF1-treated rabbits had a slower heart rate than that observed in controls. PV and LA tissues in FGF1-treated rabbits had slower beating rates and longer action potential duration than those observed in controls. Isoproterenol (1 μM)-treated PV and LA tissues in the FGF1-treated rabbits showed less changes in the increased beating rate and a lower incidence of tachypacing (20 Hz)-induced burst firing than those observed in controls. FGF1 (10 ng/mL)-treated PV and LA myocytes had less oxidative stress and Ca^2+^ transient than those observed in controls. Compared to controls, FGF1 (10 ng/mL) decreased I_Na−L_ in PV myocytes and lowered *I*_to_, *I*_Kr−tail_ in LA myocytes. Protein kinase C (PKC)ε inhibition abolished the effects of FGF1 on the ionic currents of LA and PV myocytes.

**Conclusion:**

FGF1 changes PV and LA electrophysiological characteristics possibly *via* modulating oxidative stress, Na^+^/Ca^2+^ homeostasis, and the PKCε pathway.

## Introduction

Fibroblast growth factor (FGF) 1, a signaling protein secreted mainly in the paracrine system, may be produced by cardiomyocytes (1). Animal and cellular studies have shown that FGF1 plays a cardioprotective role during ischemia and reperfusion conditions (2, 3). FGF23, a member of the FGF family, has several cardiovascular effects and can directly change cardiac electrical activity (4, 5). The overexpression of FGF1 may protect against cardiac injury through the FGF receptor (FGFR)-mediated signaling and the protein kinase C (PKC)-dependent pathway (3, 6).

Atrial fibrillation (AF) is the most common type of cardiac arrhythmia that causes heart failure and cardiovascular events (7). Enhanced trigger activity from pulmonary vein (PV) ectopic foci is critical for the genesis of AF (8, 9). Moreover, the left atrium (LA) is the most vital substrate of AF (10). The FGF1–FGFR system might play an important role in pulmonary vascular remodeling *via* chronic inflammation, fibrosis, or heart tissue repair, which has been demonstrated in obstructive lung disease (11, 12). FGF-1 protects cardiomyocytes from oxidative damage with hydrogen peroxide, which may enhance PV and atrial remodeling in the pathogenesis and perpetuation of AF (13–15). Accordingly, FGF may play a critical role in the pathogenesis of AF, and different FGF subtypes may have discrepant effects on PV and atrial arrhythmogenesis.

Calcium (Ca^2+^) regulation may induce the electrical remodeling of the PV and the atrium leading to abnormal cellular conduction properties that contribute to the pathogenesis of AF (16, 17). Moreover, the inhibition of PKC and Ca^2+^ regulatory proteins, which influence the signaling of cardiac protection *via* FGF family members, reverse electrical remodeling in FGF family member-treated PV myocytes (4, 6, 18). In addition, FGF1 can increase the cardiac expression of atrial natriuretic factor, which is blocked by PKC inhibitors (19, 20). Therefore, the purpose of this study was to investigate whether FGF1 changes the electrical properties of the AF trigger (PV) and substrate (LA) and evaluate the potential underlying mechanisms.

## Materials and Methods

### Electropharmacological Experiments in PV and LA Tissues in FGF1-Treated Rabbits

This study was approved by the local ethics review board (No. IACUC-20-365). The Male New Zealand white rabbits (2.0~3.0 kg) were received FGF1 (3 μg/kg, Sigma Aldrich, GF431) or vehicles intravenously 24 h before euthanasia by anesthetized using an intramuscular injection of xylazine hydrochloride (12 mg/kg) and inhaled overdose of isoflurane (2.0–2.5% in oxygen) from a precision vaporizer as described previously (13). The anesthesia dose was confirmed as adequate because the rabbits did not exhibit corneal reflexes and motor responses to pain stimuli induced with a scalpel tip. Electrocardiograms of the rabbits were recorded from standard lead II limb leads *via* a bio-amplifier (AD Instruments, Castle Hill, Australia), connected to a polygraph recorder (ML 845 Powerlab, AD Instruments) in a restrained condition for 6 h before euthanasia (21).

PVs and LA tissues were isolated from all rabbits after euthanasia as described previously (22). The tissue preparations were bathed in Tyrode's solution at 37°C containing 137 mM NaCl, 4 mM KCl, 15 mM NaHCO_3_, 0.5 mM NaH_2_PO_4_, 0.5 mM MgCl_2_, 2.7 mM CaCl_2_, and 11 mM dextrose. The tissues were superfused at a constant rate (3 mL/min) with Tyrode's solution, which was saturated with a gas mixture of 97% O_2_ and 3% CO_2_. The transmembrane action potentials (APs) of the PV and LA tissues were recorded using machine-pulled glass capillary microelectrodes filled with 3 M KCl, and the tissue preparations were connected to a World Precision Instrument model FD223 electrometer (FL, USA) (23). The electrical and mechanical events were simultaneously displayed on a Gould 4072 oscilloscope (OH, USA) and Gould TA11 recorder (24–26). Electrical stimuli were applied using a Grass S88 stimulator through a Grass SIU5B stimulus isolation unit.

The resting membrane potential (RMP) was measured during the period between the last repolarization and onset of the subsequent AP. The AP amplitude (APA) was obtained from the RMP to the peak of AP depolarization. The AP duration (APD) at 20, 50, and 90% repolarization of the amplitude was measured and recorded as APD_20_, APD_50_, and APD_90_, respectively. APs were analyzed for maximum upstroke velocity (dV/dt_max_), early and late diastolic depolarization (EDD and LDD) (27). Burst firing was defined as the occurrence of an accelerated spontaneous potential with sudden onset and termination. The RMP, APA, and APD were measured under spontaneous beating of PV or 2-Hz pacing of the LA tissues. The PV tissues were analyzed before and after stimulation with isoproterenol (1 μM) to observe burst firing. In addition, the LA tissues were analyzed before and after stimulation with isoproterenol (1 μM) to observe burst firing with or without high-frequency burst pacing (20 Hz) for 1 s.

### Patch Clamp Experiments in Isolated Single Cardiomyocytes Preparation

Single PV and LA cardiomyocytes were enzymatically dissociated through the same procedure described previously (28, 29). In briefly, the heart and lungs were rapidly excised following midline thoracotomy after heparin (1,000 units/kg) was intravenously administered. Proximal PVs and LA were cut away from the atrium and lung. They were gently shaken in 5~10 ml of Ca^2+^-free oxygenated Tyrode's solution until single cardiomyocytes were obtained. The solution was then gradually changed to oxygenated normal Tyrode's solution. Cells were allowed to stabilize in the bath for at least 30 min before the experiments.

Single cardiomyocytes with spontaneous activity were identified by the presence of constant beating during perfusion with Tyrode's solution. Then, single PV and LA myocytes were treated in the control and FGF1 (1 and 10 ng/mL; Sigma Aldrich, GF431) for 4–6 h are harvested for further experiments with or without PKCε inhibitor peptide (εV1-2, 200 nM, Cayman).

The whole-cell patch clamp experiment was performed in the isolated PVs and LA myocytes by using an Axopatch 200B amplifier (Axon Instruments, Foster City, CA, USA) at 35 ± 1°C (17, 30). Borosilicate glass electrodes (o.d., 1.8 mm) with a tip resistance of 3–5 MΩ were used. Before the formation of the membrane-pipette seal, the tip potentials were zeroed in Tyrode's solution. The junction potentials between the bath and pipette solution (9 mV) were corrected for the AP recordings.

APs were recorded in the current-clamp mode, and the ionic currents were recorded in the voltage-clamp mode. APs of LA myocytes were elicited in cells through brief current pulses at 1 Hz. The ionic currents were recorded at an approximately similar period (3–5 min) after rupture or perforation by amphotericin B to avoid decay of ion channel activity over time. A small hyperpolarizing step from a holding potential of −50 mV to a test potential of −55 mV for 80 ms was delivered at the beginning of each experiment. The area under the capacitative current curve was divided by the applied voltage step to calculate the total cell capacitance. Normally, series resistance (R_s_) was electronically compensated by 60–80%.

The sodium (Na^+^) current (*I*_Na_) was recorded by using 40 msec. pulses from a holding potential of −120 mV to the test potentials varying between −80 and 0 mV in 5 mV increments at a frequency of 3 Hz at room temperature (25 ± 1°C). The external solution contained: 5 mM NaCl, 133 mM CsCl, 2 mM MgCl_2_, 1.8 mM CaCl_2_, 0.002 mM nifedipine, 5 mM HEPES and 5 mM glucose (pH 7.3). Micropipettes were filled with a solution containing (in mM) 133 mM CsCl, 5 mM NaCl, 10 mM EGTA, 5 mM MgATP, 20 mM TEACl and 5 mM HEPES (pH 7.3 with CsOH).

The late Na^+^ current (*I*_Na−Late_) included a step/ramp protocol (−100 mV stepping to +20 mV for 100 ms, then ramping back to −100 mV over 100 ms) at room temperature with an external solution containing 130 mM NaCl, 5 mM CsCl, 1 mM MgCl_2_, 1 mM CaCl_2_, 10 mM HEPES, and 10 mM glucose; pH was adjusted to 7.3 using NaOH. Micropipettes were filled with a solution containing 130 mM CsCl, 4 mM Na_2_ATP, 1 mM MgCl_2_, 10 mM EGTA, and 5 mM HEPES; pH was adjusted to 7.3 using NaOH. An equilibration period for dialysis was allowed to adequately clamp the cell currents. *I*_Na−Late_ was measured as the tetrodotoxin (TTX, 30 μM)-sensitive portion of the current traces obtained when the voltage was ramped back to −100 mV (31).

The L-type Ca^2+^ current (*I*_Ca−L_) was measured as an inward current during depolarization from a holding potential of −50 mV to test potentials ranging from −40 to +60 mV in 10 mV steps for 300 ms at a frequency of 0.1 Hz using a perforated patch clamp with amphotericin B. The micropipettes were filled with a solution containing 130 mM CsCl, 1 mM MgCl_2_, 5 mM MgATP, 10 mM HEPES, 0.1 mM NaGTP, and 5 mM Na_2_ phosphocreatine, which was titrated to a pH of 7.2 using CsOH. NaCl and KCl in the external solution were replaced with tetraethylammonium chloride and CsCl, respectively. Voltage-gated Ca^2+^ channel current was plotted on the I–V curve and the curve was fitted with the modified Boltzmann equation: I (*V*) = [G_max_ × (V – V_rev_)]/{1 + e^[(V1/2−*V*)/*k*]^}, where I(*V*) is the peak current density at the command potential V, G_max_ is the maximum conductance, V_rev_ is the reverse potential, V_1/2_ is the voltage at which half-maximum *I*_Ca−L_ is observed, and *k* is the slope factor (26).

The Na^+^-Ca^2+^ exchanger (NCX) current was elicited by depolarizing pulses between −100 and +100 mV from a holding potential of −40 mV for 300 ms at a frequency of 0.1 Hz. The amplitudes of the NCX current were measured as 10-mM nickel-sensitive currents. The external solution consisted of 140 mM NaCl, 2 mM CaCl_2_, 1 mM MgCl_2_, 5 mM HEPES, and 10 mM glucose at pH 7.4 and contained 10 μM strophanthidin, 10 μM nitrendipine, and 100 μM niflumic acid. Micropipettes were filled with a solution containing 20 mM NaCl, 110 mM CsCl, 0.4 mM MgCl_2_, 1.75 mM CaCl_2_, 20 mM TEACl, 5 mM BAPTA, 5 mM glucose, 5 mM MgATP, and 10 mM HEPES (at pH 7.25 adjusted using CsOH).

The transient outward potassium (K^+^) current (*I*_to_) was studied with a double-pulse protocol. A 30-ms pre-pulse from −80 to −40 mV was used to inactivate the Na^+^ channels, followed by a 300-ms test pulse to +60 mV in 10-mV steps at a frequency of 0.1 Hz. CdCl_2_ (200 μM) was added to the bath solution to inhibit the *I*_Ca−L_. *I*_to_ was measured as the difference between the peak outward current and steady-state current.

Rapid delayed rectifier K^+^ current (*I*_Kr−tail_) was measured as the outward peak tail current density following a 3-s pre-pulse from a holding potential of −40 mV to voltage between −40 and +60 mV in 10-mV steps at a frequency of 0.1 Hz in the presence of chromanol 293B (30 μM) and CdCl_2_ (200 μM) in the Ca^2+^-free normal Tyrode's solution. Micropipettes were filled with a solution containing 120 mM KCl, 5 mM MgCl_2_, 0.36 mM CaCl_2_, 5 mM EGTA, 5 mM HEPES 5 mM glucose, 5 mM K_2_-ATP, 5 mM Na_2_-CrP, and 0.25 mM Na-GTP (at pH 7.2 adjusted using KOH).

### Measurement of Intracellular Ca^2+^ Transient

Intracellular Ca^2+^ concentration ([Ca^2+^]_i_) was recorded using a fluorometric ratio technique as previously described (26, 28). The control and FGF1 (10 ng/mL)-treated PV and LA myocytes were loaded with fluorescent Ca^2+^ (10 μM) fluo-3/AM for 30 min at room temperature. Excess extracellular dye was removed by changing the bath solution, and intracellular hydrolysis of fluo-3/AM occurred at 35 ± 1°C after 30 min. Fluo-3 fluorescence was excited with a 488-nm line of an argon ion laser. The emission was recorded at > 515 nm. The cells were repeatedly scanned at 2-ms intervals for a total duration of 6 s. Fluorescence imaging was performed using a laser scanning confocal microscope (Zeiss LSM 510, Carl Zeiss, Jena, Germany) and an inverted microscope (Axiovert 100).

Fluorescent signals were corrected for variations in dye concentrations by normalizing the fluorescence (represented by F) against baseline fluorescence (F0) to obtain reliable information about transient [Ca^2+^]_i_ changes from baseline values, as (F–F0)/F0, and to exclude variations in the fluorescence intensity by different volumes of injected dye. The [Ca^2+^]_i_ transient was measured during a 2-Hz field stimulation for 10 ms with square wave pulses that had two times the threshold strength.

### Cellular Reactive Oxygen Species Measurement

We used the CellROX green (Life Technologies, Grand Island, NY, USA) to assess cytosolic ROS production in the control and FGF1 (10 ng/mL)-treated PV and LA myocytes. Experiments were also performed using a laser scanning confocal microscope (Zeiss LSM 510, Carl Zeiss) and an inverted microscope (Axiovert 100) with a 63 × 1.25 numerical aperture oil immersion objective as described previously (32). Cardiomyocytes were maintained in oxygenated normal Tyrode's solution: 137 mM NaCl, 5.4 mM KCl, 1.8 mM CaCl_2_, 0.5 mM MgCl_2_, 10 mM HEPES and 11 mM glucose; with the pH adjusted to 7.4 by titrating with 1 N NaOH) supplemented with the appropriate fluorescent dye of 10 μM CellROX green. CellROX green was excited at 488 nm and fluorescence signals were acquired at wavelengths of > 505 nm in the XY mode of the confocal system. Fluorescent images were analyzed using Image-Pro plus 6.0 and Sigmaplot 12.3 software (4).

The level of malondialdehyde (MDA) in control and FGF1 (10 ng/mL)-treated PV and LA myocytes to detect lipid peroxidation were assessed by an ELISA kit, according to the manufacturer's guidelines and a colorimetric-fluorimetric method.

### Statistical Analysis

All quantitative data are expressed as mean ± standard deviation. The one-way analysis of variance (ANOVA) followed by Dunn's *post-hoc* test or the Wilcoxon rank-sum test was used to compare the differences between the control and FGF1-treated groups. Nominal variables were compared using Chi-squared analysis with Fisher's exact test. A *P* < 0.05 was considered statistically significant.

## Results

### Effects of FGF1 on the Electrocardiography of Rabbits and the Electrophysiological Characteristics of PV and LA Tissues

**Figure 1:**
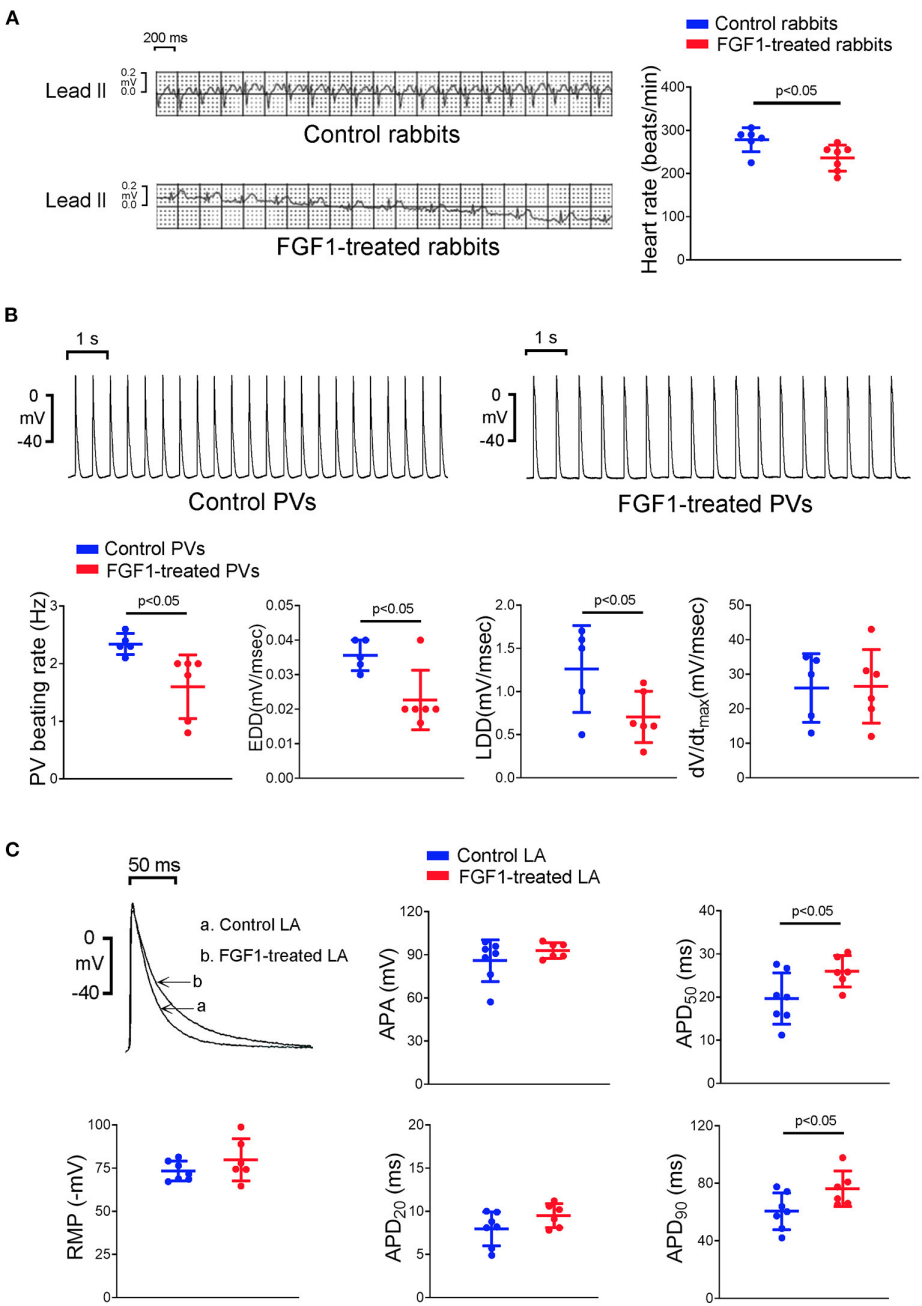
Effects of fibroblast growth factor 1 (FGF1) on the electrocardiography (ECG) of rabbits and electrophysiological characteristics of pulmonary vein (PV) and left atrial (LA) tissues. **(A)** Control rabbits (*N* = 6) had a faster regular rhythm than that observed in FGF1 (3 μg/kg)-treated rabbits (*N* = 6). **(B)** Tracings and scatter plots illustrated that PV tissues of FGF1 (3 μg/kg)-treated rabbits (*N* = 6) had a slower spontaneous activities with a decreased early and late diastolic depolarization than that observed in control rabbits (*N* = 5). **(C)** Examples and scatter plots of the action potential (AP) morphology from LA tissues under regular pacing (2 Hz) in the control (*N* = 7) and FGF1 (3 μg/kg)-treated rabbits (*N* = 6). RMP indicates resting membrane potential, APA indicates AP amplitude, APD_20_, APD_50_, and APD_90_ indicate that AP duration at 20, 50, and 90% repolarization of the amplitude, respectively, EDD indicates early diastolic depolarization, LDD indicates late diastolic depolarization, dV/dt_max_ indicates maximum upstroke velocity.

FGF1 (3 μg/kg)-treated rabbits had a slower heart rate than that observed in control rabbits (Figure 1A). PV tissues of the FGF1 (3 μg/kg)-treated rabbits had a slower beating rate than that of control rabbits. Compare to the AP of control rabbits, FGF1 (3 μg/kg)-treated rabbits had a similar dV/dt_max_, but a reduced steepness of EDD and LDD of PV tissues (Figure 1B). Moreover, LA tissues of the FGF1 (3 μg/kg)-treated rabbits had a longer APD_50_ and APD_90_ than those in control rabbits (Figure 1C).

**Figure 2:**
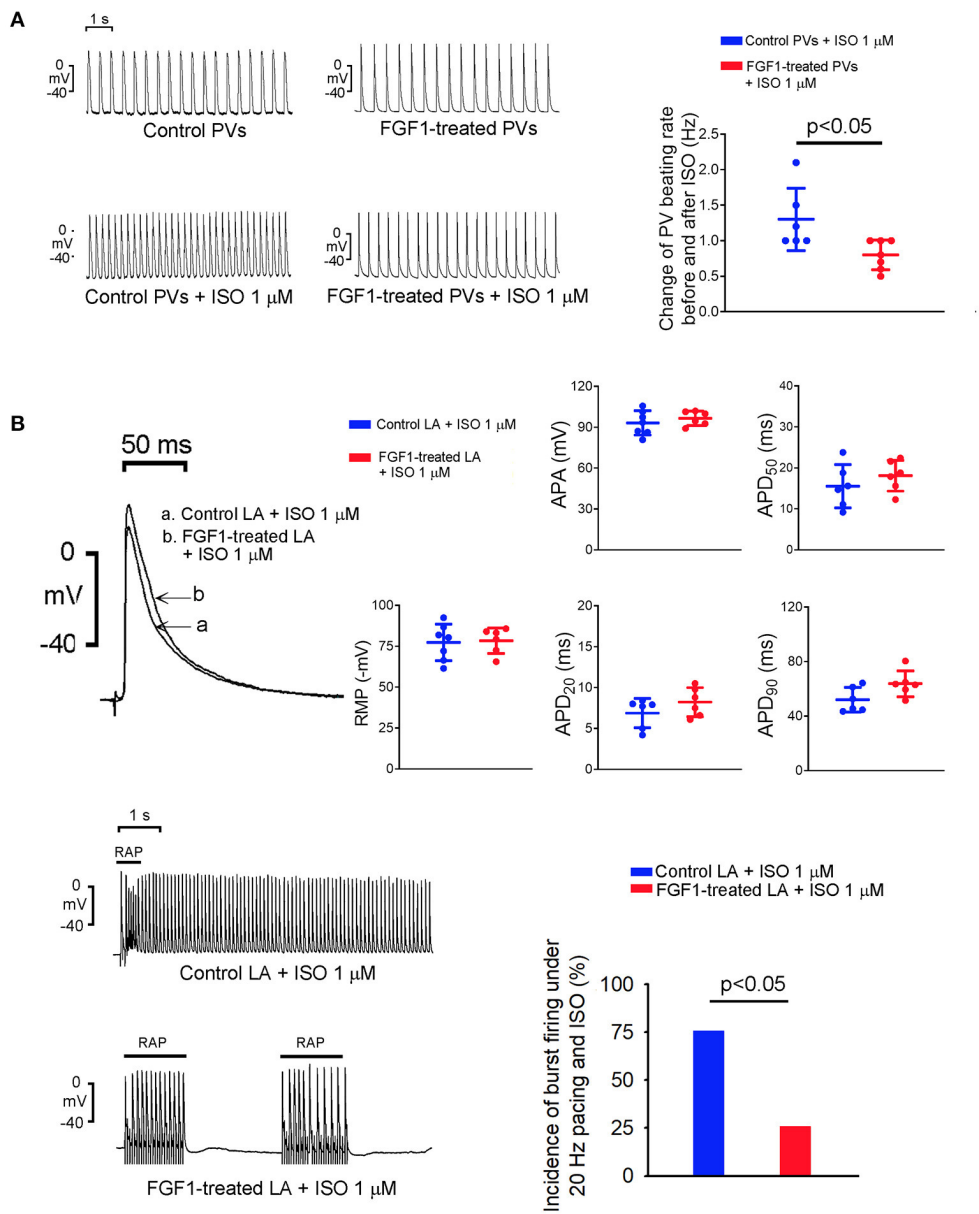
Effect of isoproterenol (ISO) on spontaneous activity of left atrium (LA) and pulmonary vein (PV) tissues with or without fibroblast growth factor 1 (FGF1) treatment. **(A)** Tracings and scatter plots of ISO (1 μM)-induced changing PV spontaneous activities in rabbits with (*N* = 7) or without (*N* = 6) FGF1 (3 μg/kg) treatment. **(B)** The upper panel shows the representative tracings and scatter plots of LA action potential morphology under regular pacing (2 Hz) in the control and FGF1 (3 μg/kg)-treated rabbits (both *N* = 6). The lower panel shows the tracings and incidence of LA burst firing under rapid atrial pacing (20 Hz) and ISO (1 μM) infusion in the control and FGF1 (3 μg/kg)-treated rabbits (both *N* = 6).

As shown in Figure 2A, isoproterenol (1 μM)-treated PV tissues in the FGF1 (3 μg/kg)-treated rabbits showed less changes in the increased beating rate than those of tissues in control rabbits. On an isoproterenol (1 μM) infusion, there were no differences in RMP, APA, APD_20_, APD_50_, and APD_90_ in LA tissues between the FGF1 (3 μg/kg)-treated and control rabbits (Figure 2B). Furthermore, the incidence of tachypacing (20 Hz)-induced burst firing in isoproterenol (1 μM)-treated LA tissues was lower in the FGF1 (3 μg/kg)-treated rabbits than in the tissues of control rabbits.

### Effects of FGF1 on the Electrophysiological Characteristics of PV and LA Myocytes

**Figure 3:**
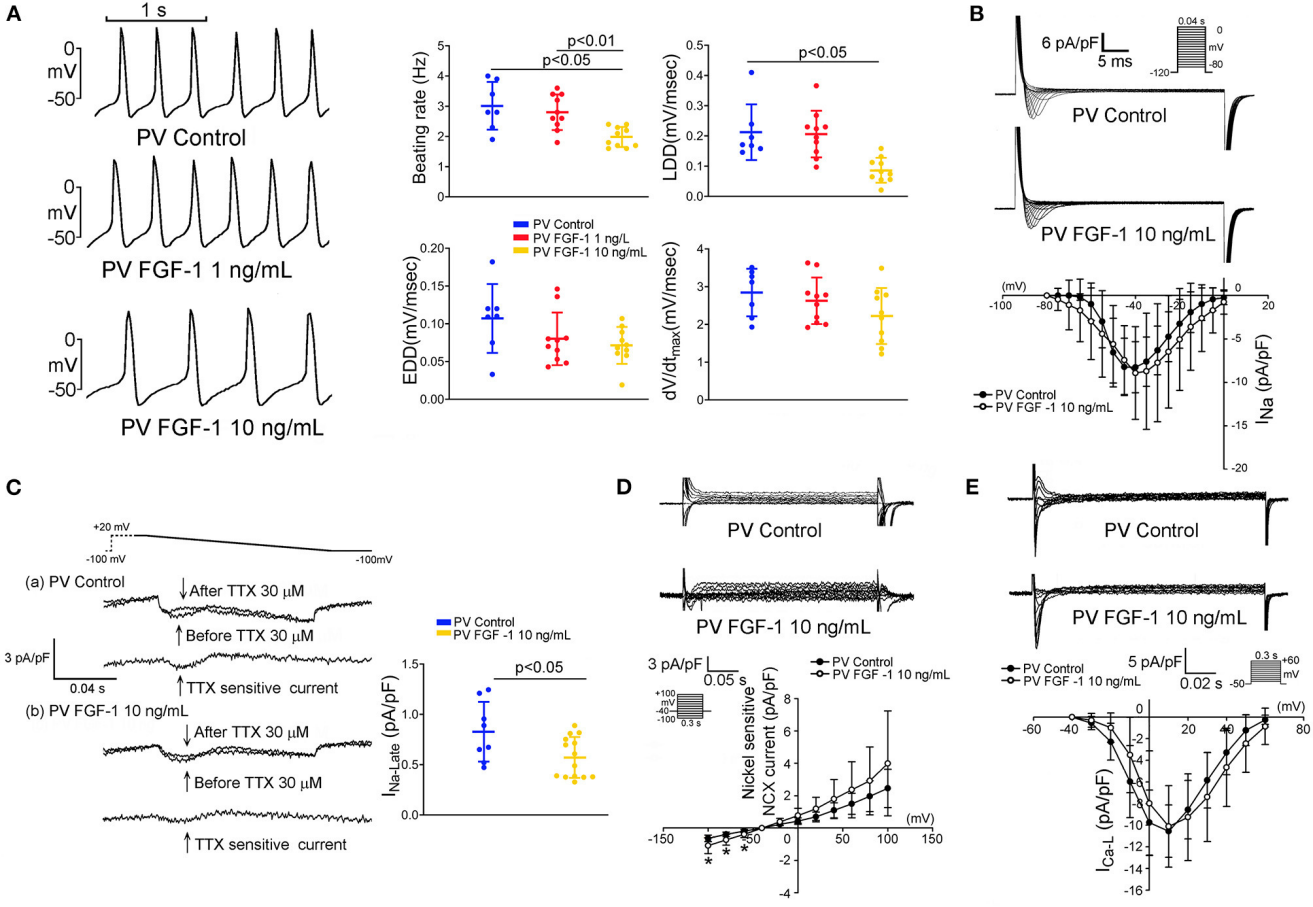
Effect of fibroblast growth factor 1 (FGF1) on spontaneous activities, sodium (Na^+^) current (*I*_Na_), late Na^+^ current (*I*_Na−Late_), sodium–calcium exchanger (NCX), and L-type calcium current (*I*_Ca−L_) of pulmonary vein (PV) myocytes. **(A)** Examples of current traces and scatter plots of beating rate in the control (*N* = 7) and FGF1 (1 and 10 ng/mL)-treated PV myocytes (both *N* = 10). **(B)** Examples of current traces and scatter plots of *I*_Na_ in the control (*N* = 8) and FGF1 (10 ng/mL)-treated PV myocytes (*N* = 9). **(C)** Examples of current traces and scatter plots of *I*_Na−Late_ in the control (*N* = 8) and FGF1 (10 ng/mL)-treated PV myocytes (*N* = 14). **(D)** Examples of current traces and *I–V* relationship of NCX in the control (*N* = 7) and FGF1 (10 ng/mL)-treated PV myocytes (*N* = 9). **(E)** Examples of current traces and *I–V* relationship of I_Ca−L_ in the control (*N* = 7) and FGF1 (10 ng/mL)-treated PV myocytes (both *N* = 9). *P < 0.05.

FGF1 (10 ng/mL)-treated PV myocytes had a slower spontaneous beating rate than that observed in control and FGF1 (1 ng/mL)-treated PV myocytes. Compared to control PV myocytes, FGF1 (3 μg/kg)-treated PV myocytes had a similar dV/dt_max_ and EDD, but a reduced steepness of LDD (Figure 3A). FGF1 (10 ng/mL)-treated PV myocytes had a smaller I_Na−L_ and larger forward mode of NCX than those observed in control myocytes (Figures 3C,D). FGF1 (10 ng/mL)-treated PV and control myocytes had similar *I*_Na_ and *I*_Ca−L_ (Figures 3B,E). The voltage-dependence of *I*_Ca−L_ activation was not different between FGF1 (1 ng/mL)-treated and control PV myocytes with V_1/2_ value of −6.77 ± 8.64 mV and −11.07 ± 7.78 mV in FGF1 (1 ng/mL)-treated and control PV myocytes, respectively.

**Figure 4:**
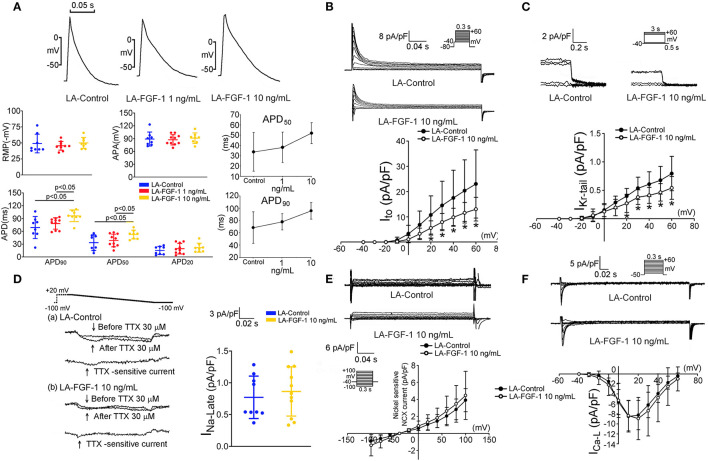
Effect of fibroblast growth factor 1 (FGF1) on action potential morphology, transient outward potassium current (*I*_to_) and rapid delayed rectifier potassium current (*I*_Kr−tail_), late sodium current (I_Na−Late_), sodium–calcium exchanger (NCX), and L-type calcium current (I_Ca−L_) in left atrium (LA) myocytes. **(A)** Examples of current traces, scatter plots of electrophysiological characteristics and dose response curves on the control (*N* = 8) and FGF1 (1 and 10 ng/mL)-treated LA myocytes (*N* = 10 and 8, respectively). **(B)** Examples of current traces and *I–V* relationship of *I*_to_ in the control (*N* = 9) and FGF1 (10 ng/mL)-treated LA myocytes (*N* = 10). **(C)** Examples of current traces and *I–V* relationship of *I*_Kr−tail_ in the control (*N* = 12) and FGF1 (10 ng/mL)-treated LA myocytes (*N* = 10). **(D)** Examples of current traces and scatter plots of *I*_Na−Late_ in the control (*N* = 9) and FGF1 (10 ng/mL)-treated LA myocytes (*N* = 11). **(E)** Examples of current traces and *I–V* relationship of NCX in the control and FGF1 (10 ng/mL)-treated LA myocytes (both *N* = 6). **(F)** Examples of current traces and *I–V* relationship of *I*_Ca−L_ in the control and FGF1 (10 ng/mL)-treated LA myocytes (both *N* = 10). *P < 0.05.

As shown in Figure 4A, FGF1 (10 ng/mL)-treated LA myocytes had longer APD_50_ and APD_90_ than those of the control and FGF1 (1 ng/mL)-treated LA myocytes. FGF1 (10 ng/mL)-treated LA myocytes had smaller *I*_to_ and *I*_Kr−tail_ than those of control myocytes (Figures 4B,C). FGF1 (10 ng/mL)-treated LA and control myocytes had similar I_Na−L_, NCX, and *I*_Ca−L_ (Figures 4D–F). The voltage-dependence of *I*_Ca−L_ activation was not different between FGF1 (1 ng/mL)-treated and control LA myocytes with V_1/2_ value of −4.44 ± 7.06 mV and −0.90 ± 3.92 mV in FGF1 (1 ng/mL)-treated and control LA myocytes, respectively.

### Effects of FGF1 on Ca^2+^ Transient and Oxidative Stress of PV and LA Myocytes

**Figure 5:**
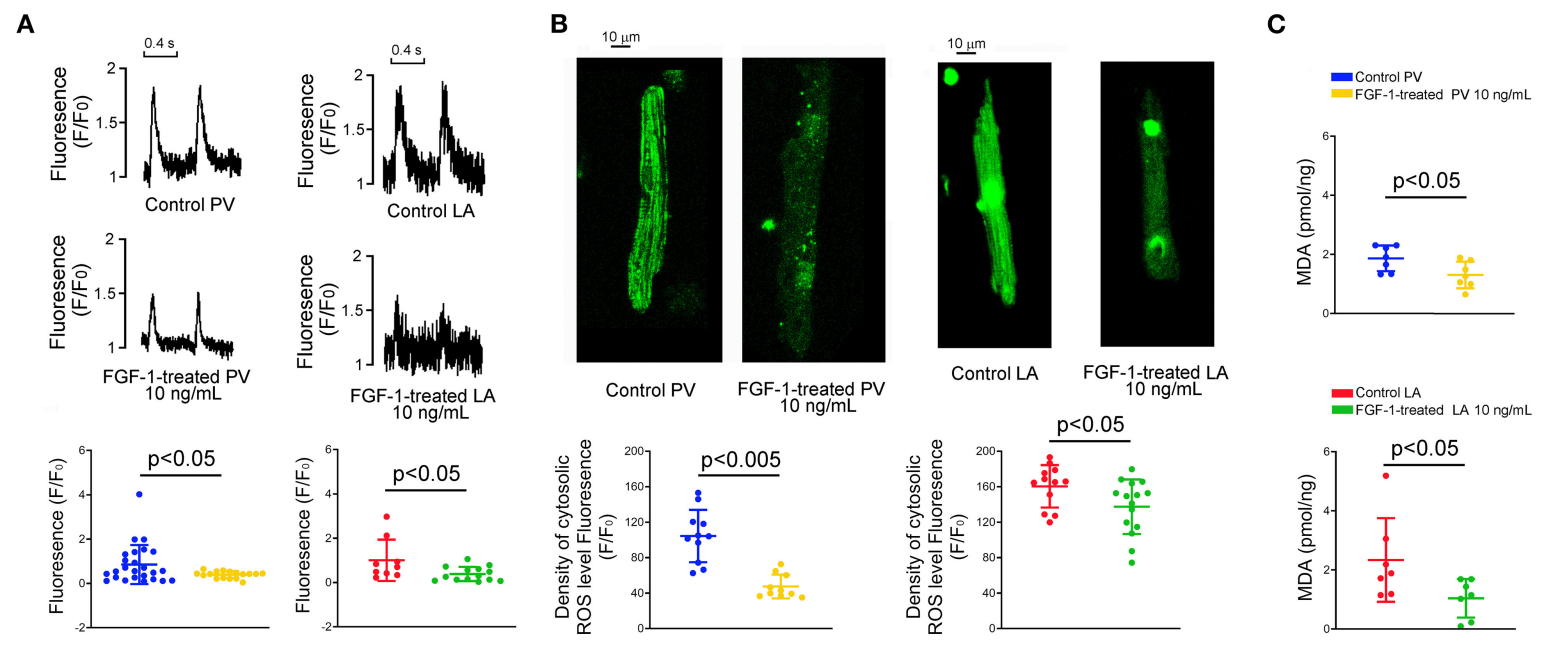
Effect of fibroblast growth factor 1 (FGF1) on intracellular calcium (Ca^2+^) homeostasis, reactive oxygen species (ROS) and cytosol malondialdehyde (MDA) in pulmonary vein (PV) and left atrium (LA) myocytes. **(A)** Examples of tracings and scatter plots of Ca^2+^ transients in the control and FGF1 (10 ng/mL)-treated PV (*N* = 25 and 17, respectively), and LA (*N* = 9 and 14, respectively), myocytes. **(B)** Examples and scatter plots of cellular levels of ROS in the control and FGF1 (10 ng/mL)-treated PV (*N* = 11 and 10, respectively), and LA (*N* = 12 and 15, respectively), myocytes. **(C)** Scatter plots of cytosol MDA in control and FGF1 (10 ng/mL)-treated PV (each *N* = 7) and LA (each *N* = 7) tissues.

FGF1 (10 ng/mL)-treated PV and LA myocytes had smaller Ca^2+^ transients than those of control PV and LA myocytes, respectively (Figure 5A). In addition, FGF1 (10 ng/mL)-treated PV and LA myocytes had less cellular oxidative stress than those of control PV and LA myocytes, respectively (Figure 5B). As shown in Figure 5C, PV and LA cells in FGF1 (3 μg/kg)-treated rabbits had less MDA compared with cells in the control rabbits.

### Effects of PKC Inhibitor on the Ionic Currents of FGF1-Treated PV and LA Myocytes

**Figure 6:**
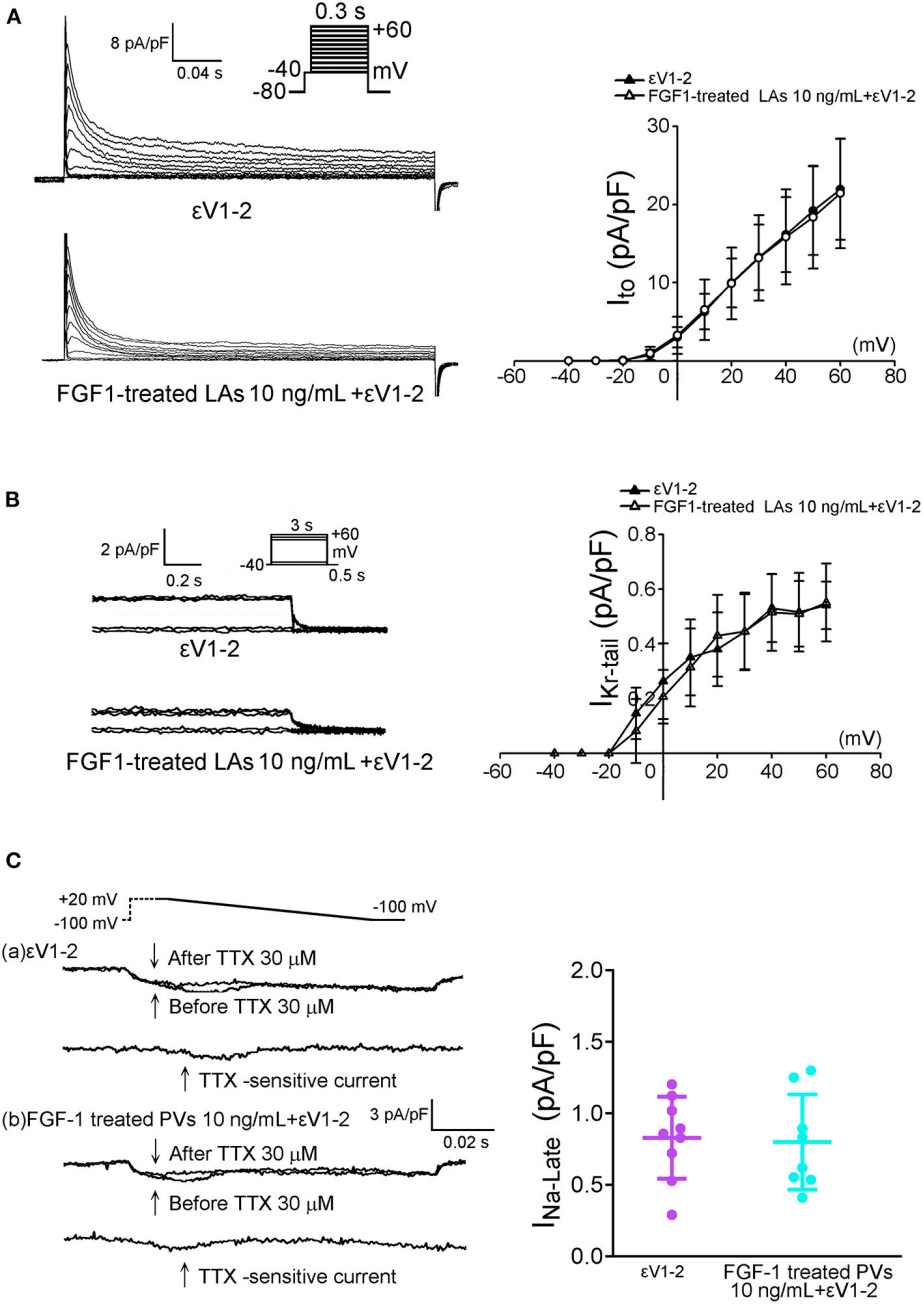
Effect of protein kinase C epsilon inhibitor (εV1-2) on transient outward potassium current (*I*_to_) and rapid delayed rectifier potassium current (*I*_Kr−tail_) in FGF1-treated left atrial (LA) myocytes, and on late sodium current (*I*_Na−Late_) in FGF1-treated pulmonary vein (PV) myocytes. **(A)** Examples of current traces and *I–V* relationship of *I*_to_ in FGF1 (10 ng/mL)-treated LA myocytes with or without εV1-2 (200 nM) (each *N* = 7). **(B)** Examples of current traces and *I–V* relationship of *I*_Kr−tail_ in FGF1 (10 ng/mL)-treated LA myocytes with (*N* = 8) or without (*N* = 9) εV1-2 (200 nM). **(C)** Examples of current traces and average data of *I*_Na−Late_ in FGF1 (10 ng/mL)-treated PV myocytes with (*N* = 8) or without (*N* = 9) εV1-2 (200 nM).

As shown in Figures 6A,B, εV1-2 (200 nM) abolished the inhibitory effect of FGF1 (10 ng/mL) on the *I*_to_ and *I*_Kr−tail_ of LA myocytes. Moreover, εV1-2 (200 nM) abolished the inhibitory effect of FGF1 (10 ng/mL) on the I_Na−L_ of PV myocytes (Figure 6C).

## Discussion

In the present study, we have demonstrated that FGF1, a potential novel therapeutic agent for treating metabolic conditions, modulates electrophysiological characteristics and possess anti-redox activity in rabbit hearts. To the best of our knowledge, this is the first study to demonstrate that FGF1 suppresses PV spontaneous activity and isoproterenol-induced LA burst firing. Moreover, FGF1 prolongs APD of the LA. *I*_Na−L_ determines the rate of spontaneous depolarization in sinoatrial node cells and contributes to cardiac automaticity (33). The effect of FGF1 on reducing the intrinsic rabbit heart rate was considered to reflect *I*_Na−L_ inhibition. *I*_Na−L_ can contribute to the diastolic depolarization of PV myocytes and the inhibition of this current suppresses PV diastolic depolarization and spontaneous APs (34). *I*_Na−L_ plays an important role in PV arrhythmogenesis and AF occurrence (4, 35). Activation of I_Na−L_ enhances the genesis of triggered activity due to Ca^2+^ overload. In the present study, FGF1 decreased the *I*_Na−L_ and increased the forward mode of NCX in PV myocytes. NCX operated dominantly in the forward mode increased Ca^2+^ efflux, which was considered a compensatory effect for decreasing intracellular Na^+^ *via* FGF1-related *I*_Na−L_ inhibition in PV myocytes. Moreover, FGF-treated PVs displayed suppression of burst firing induced by isoproterenol. Isoproterenol, an adrenergic agonist, promotes an increase in pacemaker activity and abnormal automatism through the accumulation of [Ca^2+^]_I_ (36). Ca^2+^ dysregulation may induce AF *via* the electrical remodeling of PVs and the LA (16, 17). Moreover, our previous study found that hydrogen peroxide enhances oxidative stress-induced PV and atrial arrhythmia *via* the modification of electrophysiological characteristics and Ca^2+^ or K^+^ currents (15, 37). FGF1 binding to activate all FGFRs in various tissues might regulate intracellular Ca^2+^ homeostasis leading to a protective role in metabolic disorders (38, 39). FGF1 also displays favorable effects on maintaining myocardial integrity and preventing cardiac dysfunction in post-myocardial infarction (40). Our study showed that FGF1-treated PV and LA cells have a smaller Ca^2+^ transients and less oxidative stress than those of control PV and LA cells. These results suggest that FGF1 may change the electrophysiological characteristics of PV and LA through its effects on Ca^2+^ homeostasis or ROS (4, 41). However, our study model was restricted to normal physiological conditions, and more experiments are required to understand whether FGF1 will have any antiarrhythmic effect under more complex pathological conditions (such as Ca^2+^ overload, oxidative stress, or ischemia).

APD shortening is a hallmark of AF-related electrical remodeling, likely contributing to AF maintenance and progression (42). The LA plays a critical role in AF genesis when the APD is shortened in the LA by oxidative stress or under ischemic conditions (43). Gain-of-function mutations in channel subunits generating *I*_to_ and *I*_Kr_ are associated with familial AF (44). Increased *I*_to_ and *I*_Kr_ decrease the APD and effective refractory period, thereby increasing reentry. Chauhan-Patel et al. demonstrated that long-term exposure of FGF1 in embryonic myocytes decreases K^+^ current density (45). In the present study, smaller *I*_to_ and *I*_Kr_ caused the prolongation of the APD in FGF1-treated LA myocytes, which may reduce micro-reentry arrhythmogenesis (46). Our previous study demonstrated that the enhancement of *I*_Na−L_ increases PV arrhythmogenesis (35), and selective *I*_Na−L_ inhibition suppresses the PV triggers that initiate AF (35, 47). *I*_Na−L_ inhibition by FGF1 participates in suppressing LA arrhythmogenesis.

PKC is an important enzyme involved in myocardial ischemia/reperfusion (48). Our previous study revealed that FGF23, a FGF family member related to chronic kidney disease-induced AF, increases PV arrhythmogenesis due to Na^+^ and Ca^2+^ dysregulation and mitochondrial ROS genesis *via* the activation of PKC signaling (4). In contrast, FGF1 reduced intracellular Ca^2+^ transients and oxidative stress; in addition, the effects of FGF1 on ionic currents could be blocked by PKC inhibitor in our present study. FGF1 is positively correlated with PKCε expression in cardiogenesis (49). Activation of PKC negatively regulates *I*_to_ and *I*_Kr_ in cardiomyocytes (50, 51). Moreover, PKCε activation induces the synthesis of NOS-derived nitric oxide and regulates cardiac intracellular Na^+^ and Ca^2+^, which plays an important role in protecting the heart against Na^+^ and Ca^2+^ overload (52). It is speculated that FGF1 may decrease ROS levels and modulate PKC activity that may change PV and LA electrophysiological characteristics (48, 53).

## Conclusion

In this study, we found that FGF1 can modulate PV and LA electrophysiological characteristics and Ca^2+^ homeostasis through the suppression of oxidative stress-induced PKC activation.

## Data Availability Statement

The raw data supporting the conclusions of this article will be made available by the authors, without undue reservation.

## Ethics Statement

The animal study was reviewed and approved by National Defense Medical Center Institutional Animal Care and Use Committee. Written informed consent was obtained from the owners for the participation of their animals in this study.

## Author Contributions

Y-YL and S-YH conducted experiments, analyzed data, and wrote the manuscript. Y-CC and Y-HK conducted experiments. Y-KL and SH contributed to the analysis and interpretation of the data. C-CC analyzed data and revised the manuscript. S-AC and Y-JC conceived and designed the study, revised the manuscript, and gave final approval. All authors have read and approved the final manuscript.

## Funding

This work was supported by grants from the Ministry of Science and Technology (MOST108-2314-B-281-007-MY3, MOST109-2314-B-038-124-MY3, MOST109-2314-B-016-045, MOST109-2314-B-016-001-MY2, MOST110-2314-B-038-107-MY3, and MOST110-2314-B-016-037-MY3), Taipei Medical University-Wan Fang Hospital (106-swf-10, 107-wf-swf-02, 107-wf-swf-07, 107-wf-eva-13, 108-wf-eva-06, 108-wf-swf-01, 108-wf-swf-06, 109-wf-eva-04, 109-wf-eva-18, and 109-wf-swf-09), the Chi-Mei Medical Center (105CM-TMU-13, 106CM-TMU-08, 108CM-TMU-05, and 110CM-TMU-11), Ministry of National Defense-Medical Affairs Bureau (MND-MAB-D-111105), and the Foundation for the Development of Internal Medicine in Okinawa (2-02-005).

## Conflict of Interest

The authors declare that the research was conducted in the absence of any commercial or financial relationships that could be construed as a potential conflict of interest.

## Publisher's Note

All claims expressed in this article are solely those of the authors and do not necessarily represent those of their affiliated organizations, or those of the publisher, the editors and the reviewers. Any product that may be evaluated in this article, or claim that may be made by its manufacturer, is not guaranteed or endorsed by the publisher.

## References

[1] Itoh N Ohta H. Pathophysiological roles of FGF signaling in the heart. Front Physiol. (2013) 4:247. 10.3389/fphys.2013.00247 24046748 PMC3764331

[2] Cuevas P Reimers D Carceller F Martinez-Coso V Redondo-Horcajo M Saenz de Tejada I. Fibroblast growth factor-1 prevents myocardial apoptosis triggered by ischemia reperfusion injury. Eur J Med Res. (1997) 2:465–8. 9385115

[3] Htun P Ito WD Hoefer IE Schaper J Schaper W. Intramyocardial infusion of FGF-1 mimics ischemic preconditioning in pig myocardium. J Mol Cell Cardiol. (1998) 30:867–77. 10.1006/jmcc.1998.0654 9602436

[4] Huang SY Chen YC Kao YH Hsieh MH Lin YK Chung CC. Fibroblast growth factor 23 dysregulates late sodium current and calcium homeostasis with enhanced arrhythmogenesis in pulmonary vein cardiomyocytes. Oncotarget. (2016) 7:69231–42. 10.18632/oncotarget.12470 27713141 PMC5342473

[5] Kao YH Chen YC Lin YK Shiu RJ Chao TF Chen SA. FGF-23 dysregulates calcium homeostasis and electrophysiological properties in HL-1 atrial cells. Eur J Clin Invest. (2014) 44:795–801. 10.1111/eci.12296 24942561

[6] Palmen M Daemen MJ De Windt LJ Willems J Dassen WR Heeneman S. Fibroblast growth factor-1 improves cardiac functional recovery and enhances cell survival after ischemia and reperfusion: a fibroblast growth factor receptor, protein kinase C, and tyrosine kinase-dependent mechanism. J Am Coll Cardiol. (2004) 44:1113–23. 10.1016/j.jacc.2004.05.067 15337227

[7] Kannel WB Wolf PA Benjamin EJ Levy D. Prevalence, incidence, prognosis, and predisposing conditions for atrial fibrillation: population-based estimates. Am J Cardiol. (1998) 82:2N–9. 10.1016/S0002-9149(98)00583-9 9809895

[8] Chen SA Hsieh MH Tai CT Tsai CF Prakash VS Yu WC. Initiation of atrial fibrillation by ectopic beats originating from the pulmonary veins: electrophysiological characteristics, pharmacological responses, and effects of radiofrequency ablation. Circulation. (1999) 100:1879–86. 10.1161/01.CIR.100.18.1879 10545432

[9] Chen YC Pan NH Cheng CC Higa S Chen YJ Chen SA. Heterogeneous expression of potassium currents and pacemaker currents potentially regulates arrhythmogenesis of pulmonary vein cardiomyocytes. J Cardiovasc Electrophysiol. (2009) 20:1039–45. 10.1111/j.1540-8167.2009.01480.x 19473300

[10] Lo LW Tai CT Lin YJ Chang SL Wongcharoen W Chang SH. Progressive remodeling of the atrial substrate–a novel finding from consecutive voltage mapping in patients with recurrence of atrial fibrillation after catheter ablation. J Cardiovasc Electrophysiol. (2007) 18:258–65. 10.1111/j.1540-8167.2007.00719.x 17241372

[11] Szebenyi G Fallon JF. Fibroblast growth factors as multifunctional signaling factors. Int Rev Cytol. (1999) 185:45–106. 10.1016/S0074-7696(08)60149-7 9750265

[12] Kranenburg AR De Boer WI Van Krieken JH Mooi WJ Walters JE Saxena PR. Enhanced expression of fibroblast growth factors and receptor FGFR-1 during vascular remodeling in chronic obstructive pulmonary disease. Am J Respir Cell Mol Biol. (2002) 27:517–25. 10.1165/rcmb.4474 12397010

[13] Li XK Lin ZF Li Y Hu S Tan Y Huang Z. Cardiovascular protection of nonmitogenic human acidic fibroblast growth factor from oxidative damage *in vitro* and *in vivo*. Cardiovasc Pathol. (2007) 16:85–91. 10.1016/j.carpath.2006.11.004 17317541

[14] Lin YK Lin FZ Chen YC Cheng CC Lin CI Chen YJ. Oxidative stress on pulmonary vein and left atrium arrhythmogenesis. Circ J. (2010) 74:1547–56. 10.1253/circj.CJ-09-0999 20562495

[15] Huang SY Lu YY Chen YC Chen WT Lin YK Chen SA. Hydrogen peroxide modulates electrophysiological characteristics of left atrial myocytes. Acta Cardiol Sin. (2014) 30:38–45. 27122766 PMC4804819

[16] Lo LW Chen YC Chen YJ Wongcharoen W Lin CI Chen SA. Calmodulin kinase II inhibition prevents arrhythmic activity induced by alpha and beta adrenergic agonists in rabbit pulmonary veins. Eur J Pharmacol. (2007) 571:197–208. 10.1016/j.ejphar.2007.05.066 17612522

[17] Suenari K Chen YC Kao YH Cheng CC Lin YK Chen YJ. Discrepant electrophysiological characteristics and calcium homeostasis of left atrial anterior and posterior myocytes. Basic Res Cardiol. (2011) 106:65–74. 10.1007/s00395-010-0132-1 21072524

[18] Padua RR Merle PL Doble BW Yu CH Zahradka P Pierce GN. FGF-2-induced negative inotropism and cardioprotection are inhibited by chelerythrine: involvement of sarcolemmal calcium-independent protein kinase C. J Mol Cell Cardiol. (1998) 30:2695–709. 10.1006/jmcc.1998.0832 9990540

[19] Tokola H Salo K Vuolteenaho O Ruskoaho H. Basal and acidic fibroblast growth factor-induced atrial natriuretic peptide gene expression and secretion is inhibited by staurosporine. Eur J Pharmacol. (1994) 267:195–206. 10.1016/0922-4106(94)90171-6 7519562

[20] Rockman HA Ross RS Harris AN Knowlton KU Steinhelper ME Field LJ. Segregation of atrial-specific and inducible expression of an atrial natriuretic factor transgene in an *in vivo* murine model of cardiac hypertrophy. Proc Natl Acad Sci USA. (1991) 88:8277–81. 10.1073/pnas.88.18.8277 1832775 PMC52490

[21] Lee TI Kao YH Chen YC Pan NH Lin YK Chen YJ. Cardiac peroxisome-proliferator-activated receptor expression in hypertension co-existing with diabetes. Clin Sci (Lond). (2011) 121:305–12. 10.1042/CS20100529 21501116

[22] Huang SY Chen YC Kao YH Hsieh MH Chen YA Chen WP. Renal failure induces atrial arrhythmogenesis from discrepant electrophysiological remodeling and calcium regulation in pulmonary veins, sinoatrial node, and atria. Int J Cardiol. (2016) 202:846–57. 10.1016/j.ijcard.2015.10.004 26476981

[23] Chang SL Chen YC Chen YJ Wangcharoen W Lee SH Lin CI. Mechanoelectrical feedback regulates the arrhythmogenic activity of pulmonary veins. Heart. (2007) 93:82–8. 10.1136/hrt.2006.089359 16905626 PMC1861344

[24] Wongcharoen W Chen YC Chen YJ Chang CM Yeh HI Lin CI. Effects of a Na+/Ca^2+^ exchanger inhibitor on pulmonary vein electrical activity and ouabain-induced arrhythmogenicity. Cardiovasc Res. (2006) 70:497–508. 10.1016/j.cardiores.2006.02.026 16574085

[25] Chen YJ Chen SA Chang MS Lin CI. Arrhythmogenic activity of cardiac muscle in pulmonary veins of the dog: implication for the genesis of atrial fibrillation. Cardiovasc Res. (2000) 48:265–73. 10.1016/S0008-6363(00)00179-6 11054473

[26] Lu YY Chung FP Chen YC Tsai CF Kao YH Chao TF. Distinctive electrophysiological characteristics of right ventricular out-flow tract cardiomyocytes. J Cell Mol Med. (2014) 18:1540–8. 10.1111/jcmm.12329 24913286 PMC4190900

[27] Huang JH Chen YC Lu YY Lin YK Chen SA Chen YJ. Arginine vasopressin modulates electrical activity and calcium homeostasis in pulmonary vein cardiomyocytes. J Biomed Sci. (2019) 26:71. 10.1186/s12929-019-0564-3 31530276 PMC6747756

[28] Huang SY Chen YC Kao YH Hsieh MH Lin YK Chen SA. Redox and activation of protein kinase a dysregulates calcium homeostasis in pulmonary vein cardiomyocytes of chronic kidney disease. J Am Heart Assoc. (2017) 6:e005701. 10.1161/JAHA.117.005701 28701305 PMC5586294

[29] Lin YK Chen YC Huang JH Lin YJ Huang SS Chen SA. Leptin modulates electrophysiological characteristics and isoproterenol-induced arrhythmogenesis in atrial myocytes. J Biomed Sci. (2013) 20:94. 10.1186/1423-0127-20-94 24354396 PMC3878176

[30] Chen YC Kao YH Huang CF Cheng CC Chen YJ Chen SA. Heat stress responses modulate calcium regulations and electrophysiological characteristics in atrial myocytes. J Mol Cell Cardiol. (2010) 48:781–8. 10.1016/j.yjmcc.2009.08.006 19695257

[31] Orth PM Hesketh JC Mak CK Yang Y Lin S Beatch GN. RSD1235 blocks late INa and suppresses early afterdepolarizations and torsades de pointes induced by class III agents. Cardiovasc Res. (2006) 70:486–96. 10.1016/j.cardiores.2006.01.026 16545351

[32] Viatchenko-Karpinski S Kornyeyev D El-Bizri N Budas G Fan P Jiang Z. Intracellular Na+ overload causes oxidation of CaMKII and leads to Ca2+ mishandling in isolated ventricular myocytes. J Mol Cell Cardiol. (2014) 76:247–56. 10.1016/j.yjmcc.2014.09.009 25252177 PMC4250389

[33] Huang X Du Y Yang P Lin S Xi Y Yang Z. Age-dependent alterations of voltage-gated Na(+) channel isoforms in rat sinoatrial node. Mech Ageing Dev. (2015) 152:80–90. 10.1016/j.mad.2015.10.003 26528804

[34] Song Y Shryock JC Belardinelli L. A slowly inactivating sodium current contributes to spontaneous diastolic depolarization of atrial myocytes. Am J Physiol Heart Circ Physiol. (2009) 297:H1254–62. 10.1152/ajpheart.00444.2009 19700626

[35] Lu YY Cheng CC Chen YC Chen SA Chen YJ. ATX-II-induced pulmonary vein arrhythmogenesis related to atrial fibrillation and long QT syndrome. Eur J Clin Invest. (2012) 42:823–31. 10.1111/j.1365-2362.2012.02655.x 22339387

[36] Chen YJ Chen SA Chen YC Yeh HI Chan P Chang MS. Effects of rapid atrial pacing on the arrhythmogenic activity of single cardiomyocytes from pulmonary veins: implication in initiation of atrial fibrillation. Circulation. (2001) 104:2849–54. 10.1161/hc4801.099736 11733406

[37] Hanafy DA Chen YC Chang SL Lu YY Lin YK Kao YH. Different effects of dronedarone and amiodarone on pulmonary vein electrophysiology, mechanical properties and H2O2-induced arrhythmogenicity. Eur J Pharmacol. (2013) 702:103–8. 10.1016/j.ejphar.2013.01.037 23376158

[38] Nies VJ Sancar G Liu W van Zutphen T Struik D Yu RT. Fibroblast growth factor signaling in metabolic regulation. Front Endocrinol. (2015) 6:193. 10.3389/fendo.2015.00193 26834701 PMC4718082

[39] Eswarakumar VP Lax I Schlessinger J. Cellular signaling by fibroblast growth factor receptors. Cytokine Growth Factor Rev. (2005) 16:139–49. 10.1016/j.cytogfr.2005.01.001 15863030

[40] Huang C Liu Y Beenken A Jiang L Gao X Huang Z. A novel fibroblast growth factor-1 ligand with reduced heparin binding protects the heart against ischemia-reperfusion injury in the presence of heparin co-administration. Cardiovasc Res. (2017) 113:1585–602. 10.1093/cvr/cvx165 29016740 PMC5852627

[41] Lu YY Lin YK Wen ZH Chen YC Chen SA Chen YJ. Latrunculin B modulates electrophysiological characteristics and arrhythmogenesis in pulmonary vein cardiomyocytes. Clin Sci. (2016) 130:721–32. 10.1042/CS20150593 26839418

[42] Heijman J Voigt N Nattel S Dobrev D. Cellular and molecular electrophysiology of atrial fibrillation initiation, maintenance, and progression. Circ Res. (2014) 114:1483–99. 10.1161/CIRCRESAHA.114.302226 24763466

[43] Chan CS Lin YK Kao YH Chen YC Chen SA Chen YJ. Hydrogen sulphide increases pulmonary veins and atrial arrhythmogenesis with activation of protein kinase C. J Cell Mol Med. (2018) 22:3503–13. 10.1111/jcmm.13627 29659148 PMC6010708

[44] Christophersen IE Ellinor PT. Genetics of atrial fibrillation: from families to genomes. J Hum Genet. (2016) 61:61–70. 10.1038/jhg.2015.44 25994868

[45] Chauhan-Patel R Spruce AE. Differential regulation of potassium currents by FGF-1 and FGF-2 in embryonic Xenopus laevis myocytes. J Physiol. (1998) 512:109–18. 10.1111/j.1469-7793.1998.109bf.x 9729621 PMC2231171

[46] Chen WT Chen YC Hsieh MH Huang SY Kao YH Chen YA. The uremic toxin indoxyl sulfate increases pulmonary vein and atrial arrhythmogenesis. J Cardiovasc Electrophysiol. (2015) 26:203–10. 10.1111/jce.12554 25244538

[47] Burashnikov A Antzelevitch C. Role of late sodium channel current block in the management of atrial fibrillation. Cardiovasc Drugs Ther. (2013) 27:79–89. 10.1007/s10557-012-6421-1 23108433 PMC3557765

[48] Teng JC Kay H Chen Q Adams JS Grilli C Guglielmello G. Mechanisms related to the cardioprotective effects of protein kinase C epsilon (PKC epsilon) peptide activator or inhibitor in rat ischemia/reperfusion injury. Naunyn Schmiedebergs Arch Pharmacol. (2008) 378:1–15. 10.1007/s00210-008-0288-5 18496674

[49] Lin HY Lee DC Wang HD Chi YH Chiu IM. Activation of FGF1B promoter and FGF1 are involved in cardiogenesis through the signaling of PKC, but Not MAPK. Stem Cells Dev. (2015) 24:2853–63. 10.1089/scd.2015.0157 26414172

[50] Niwa N Nerbonne JM. Molecular determinants of cardiac transient outward potassium current (Ito) expression and regulation. J Mol Cell Cardiol. (2010) 48:12–25. 10.1016/j.yjmcc.2009.07.013 19619557 PMC2813406

[51] Sutherland-Deveen ME Wang T Lamothe SM Tschirhart JN Guo J Li W. Differential regulation of human ether-a-go-go-related gene (hERG) current and expression by activation of protein kinase C. Mol Pharmacol. (2019) 96:1–12. 10.1124/mol.118.115188 31015282

[52] Pavlovic D Hall AR Kennington EJ Aughton K Boguslavskyi A Fuller W. Nitric oxide regulates cardiac intracellular Na+ and Ca^2+^ by modulating Na/K ATPase via PKCepsilon and phospholemman-dependent mechanism. J Mol Cell Cardiol. (2013) 61:164–71. 10.1016/j.yjmcc.2013.04.013 23612119 PMC3981027

[53] Dhalla NS Muller AL. Protein kinases as drug development targets for heart disease therapy. Pharmaceuticals. (2010) 3:2111–45. 10.3390/ph3072111 27713345 PMC4036665

